# Topical and Intralesional Immunotherapy for the Management of Skin Cancer in Special Locations: Lips and Eyelids

**DOI:** 10.3390/cancers15205018

**Published:** 2023-10-17

**Authors:** Cecilia Buján Bonino, Isabel Rodríguez-Blanco, Dolores Sánchez-Aguilar Rojas, Hugo A. Vázquez Veiga, Ángeles Flórez

**Affiliations:** 1 Department of Dermatology, University Hospital of Santiago de Compostela, 36001 Santiago de Compostela, Spain; 2 Department of Dermatology, University Hospital of Pontevedra, 36162 Pontevedra, Spain; 3 DIPO Research Group, Galicia Sur Health Research Institute (IIS Galicia Sur), Servizo Galego de Saúde—Universidade de Vigo (SERGAS—UVIGO), 36213 Vigo, Spain

**Keywords:** periocular, perioral, eyelid, lip, skin cancer, topical, intralesional, immunotherapy

## Abstract

**Simple Summary:**

This article discusses the use of topical and intralesional immunotherapy for treating skin cancers in sensitive areas like the lips and eyelids. Surgical options might not be suitable due to potential deformities. This study reviews experiences with various topical and intralesional therapies, such as imiquimod, 5-fluorouracil (5-FU), photodynamic therapy (PDT), ingenol mebutate (IM), diclofenac, intralesional methotrexate, and interferon. While the evidence is limited due to varied studies and few clinical trials, these treatments show promise with high response rates and minimal side effects for specific cases.

**Abstract:**

The use of topical and intralesional immunotherapy in the treatment of cutaneous malignant neoplasia in sensitive areas such as the lips and eyelids is discussed. Surgery may not be feasible or may result in deformities in these areas, making alternative treatment options necessary. A narrative literature review was conducted using MEDLINE (PubMed) as the main literature database, collecting available evidence of experiences with various topical and intralesional therapies in the aforementioned anatomical locations, ranging from case reports to clinical trials. The clearance rates and potential adverse reactions of therapeutic options such as imiquimod 5%, 5-fluorouracil (5-FU), photodynamic therapy (PDT), ingenol mebutate (IM), diclofenac, intralesional methotrexate, and interferon are reviewed. Although limited by their heterogeneity and the scarcity of clinical trials, these studies point towards promising response rates and minimal adverse effects, making these treatments viable options in selected cases.

## 1. Introduction, Materials, and Methods

While surgery remains the mainstay of treatment for cutaneous malignant neoplasias, it can be deforming and/or unfeasible in functionally and aesthetically sensitive areas of the facial anatomy, such as the lips and eyelids. Other therapeutic options, such as radiotherapy, immunotherapy, or targeted therapy, are tissue-sparing but potentially locally or systemically aggressive. The use of well-known antitumoral local therapies with minimal adverse effects (AEs) has been specifically described in the most common neoplasia in these locations; however, the evidence is heterogeneous and consists mainly of a series of cases.

A narrative literature review was performed to analyse the available non-surgical treatment modalities for the management of perioral and periocular cutaneous cancer, as well as their clearance rates and potential adverse effects. The applied inclusion criteria were as follows: (1) Articles pertaining the topical, intralesional, or intraarterial treatment of any cutaneous cancer type in the aforementioned anatomical locations. (2) Articles describing the systemic administration of any agent intended for local activation (i.e., intravenous photosensitizer in photodynamic therapy). (3) No restrictions were applied regarding previous or concomitant treatments. (4) No exclusions were carried out based on article type. Case reports were included. (5) Manuscripts in languages other than English or Spanish were not considered.

MEDLINE (PubMed) was the main literature database used for this research, which was conducted in April 2023 using different search strategies and combinations around keywords describing location (“periocular”, “eyelid”, “lip”), neoplastic type (using both general terms such as “cancer” and “skin cancer” or more specific terms such as “basal cell carcinoma”, “squamous cell neoplasia”, “melanoma”, or “lentigo maligna”), and treatments (both general terms such as “topical” or “intralesional” and research directed at specific modalities such as “imiquimod”, “5-fluorouracil”, or “photodynamic therapy”).

A review was executed using a three-phase method. In the first step, a large article pool was retrieved among the research results based on their titles. In a second phase, articles were screened by their abstracts, excluding those deemed to be not relevant according to the set inclusion criteria. Finally, an analysis of the selected articles and their references was undertaken, and the main findings were qualitatively summarized.

Data on the studies included were compiled and tabulated.

## 2. Topical Immunotherapy in Perioral Cutaneous Cancer

Squamous cell carcinoma (SCC) is the most common neoplasia affecting the lip. This hazardous tumoral type is fourfold more prone to distant involvement than SCC in other locations [1] as well as local recurrences [2]. It represents up to 90% of labial malignant tumours, and it is located on the lower lip in more than 80% of cases [3], due its higher exposure to UV radiation, the pivotal factor in its pathogenesis. Other drivers in its progression are human papillomavirus infection (25% of cases) [4], immunosuppression [3], and the use of tobacco and alcohol [3].

### 2.1. Actinic Cheilitis

Lip SCC is preceded in 90% by actinic cheilitis (AC), clinically characterized by scaling, lip atrophy, loss of vermillion borders, ulceration, and crusting [5,6]. AC represents local neoplastic changes which can eventually develop into lip SCC. Advanced age, tobacco use, a lighter Fitzpatrick skin type, and a story of outdoor working have all been shown to positively correlate with the development of AC [7]. An epidemiological study found a prevalence of AC of up to 31.3% of those older than 45 [8]. The risk of SCC developing in AC is 2.5 times higher than it is for actinic keratoses (AK) in other areas [3]. Therefore, its active treatment as a neoplasia in situ is of utmost importance in the prevention of SCC.

Radical treatment modalities, such as vermilionectomy and CO_2_ laser, have shown almost total complete response (CR) rates with very rare recurrences (100% in the case of surgery with no recurrences and CR of 93.39% with a recurrence rate of 6.42% in the case of laser in a systematic review) [9]. However, these procedures and their postoperative periods are painful and pose a risk of aesthetically undesirable scarring, prompting the search for other less aggressive and reasonably effective treatment options.

Imiquimod 5% has been successfully used in the management of AC. It acts as an agonist on Toll-like receptor 7, stimulating the innate immune system with the subsequent release of interferon and proinflammatory cytokines [6]. A systematic review showed a CR of 76% after a follow-up time of up to 18 months, with only transient local irritation as an AE [9]. Another systematic review stated a clinical CR rate of 73.3%, but only two out of the five cases in which the histologic response was studied showed complete clearance [10]. However, the histological response in 73% of patients was also reported in another study [6]. Different research comparing imiquimod 5% (thrice a week, 4 weeks) to other local therapies for AC showed a total response rate of 90% (CR in 50%) without recurrences in a minimal follow-up time of 6 months, resulting in a moderate inflammatory reaction [1,5]. Overall, despite the scarce evidence available, it is concluded that imiquimod 5% shows a favourable response rate, albeit with some degree of discomfort [5].

5-Fluorouracil (5-FU) 1% and 5% is also a widely used agent in the management of actinic damage, which was shown to lie amongst the most beneficial treatments in other areas [11]. It blocks the synthesis of DNA through the inhibition of thymidylate synthase [6]. In AC, an overall CR rate of 75% was achieved in a systematic review. However, the same study found clinical recurrence in 31.8% of cases, with only a partial histological response in five out of six cases studied. Moreover, treatment was discontinued in 10% of cases due to local AEs [9]. Another systematic review with a narrower scope noted that 5-FU was the only treatment with associated recurrences [5].

Photodynamic therapy (PDT) has been widely described as a treatment for AC, using different photosensitizers and light sources. It works by not only inducing direct cell death through apoptosis and necrosis but also triggering both innate and adaptative immune responses through the liberation of damage-associated molecular patterns (DAMPs) and the creation of new antigens, among others [12]. A systematic review including 241 patients treated with conventional or daylight PDT found a CR rate of 66.67% with 14.07% of recurrences. It should be highlighted that response rates were higher with aminolevulinic acid (ALA) than with methylaminolevulinate (MAL), both on clinical (CR 73.5% vs. 63.8%) and histological (53.2% vs. 23.4%) grounds [9,10].

It has been suggested that daylight PDT (DLPDT) might be non-inferior to conventional PDT and better tolerated. In two systematic reviews, MAL-DLPDT provided the best clinical results within the modality, with a CR in 82.6% of patients [9,10]. Another study found that 16 out of 20 patients who received MAL-DLPDT for the treatment of AC and completed follow-up were disease-free after a year [13]. Treatment was painless in 80% of cases [13]. A series of 11 patients treated with repeated DLPDT sessions found clinical and histological CR rates over 90% after a mean of 2.7 sessions, and it was proposed that response rates may be higher than previously described when the number of sessions is not systematically limited to two [14].

Laser-mediated PDT, using both Er:YAG and pulsed dye, showed a CR in 75.5% and recurrences in 6.1% of patients in a review when considered as a whole [9]. However, a trial comparing Er:YAG MAL-PDT with conventional MAL-PDT described greater differences in both efficacy (92% vs. 59%) and recurrence rates (8% vs. 50%), favouring the first, with no differences regarding cosmetic results [15]. PDT plus imiquimod 5%, administered as two PDT sessions two weeks apart followed by imiquimod applied thrice a week for four weeks, was found to have CR rates of 79.4%, superior to those for conventional PDT alone and slightly higher than those reported for laser-mediated PDT. It is suggested that post-PDT inflammation promotes the recruitment of innate immunity induced by imiquimod [10,16].

Ingenol mebutate (IM) is a vegetal derivative able to induce cell death through the Hedgehog pathway [6]. It had a CR rate of 41.5% with no described recurrences [9]. A series of seven patients found a CR in three patients and a satisfactory clinical response of at least 75% of improvement from baseline in all of the remainder [17]. Another series of 14 patients did not find a clinical nor histological CR, but every patient attained some degree of clinical improvement without major complications [18]. Another instance compares IM to imiquimod and diclofenac, finding a CR rate of 40% for IM, slightly lower than that of imiquimod, 50%, and higher than that of diclofenac, 20%. It concludes that that IM is a useful option in those patients with adherence difficulties due to its shorter posology (three consecutive days) [1].

However, in a systematic review, diclofenac showed a CR rate of 45.1% with histological resolutions in two-thirds of cases assessed. The cosmetic results were deemed to be excellent in 100% of cases [9]. Another review described various instances in which diclofenac was found to be effective and to produce little irritation, suggesting that it may be especially useful in less hyperkeratotic, eroded cases of AC in which its absorption is optimal [5].

### 2.2. Invasive Squamous Cell Carcinoma

The use of topical therapies in invasive SCC of the lip has been less thoroughly reported, limited to scarce case reports, usually as adjuvants. Laser-mediated ALA-PDT has been employed after debulking surgery in three cases of lip SCC, as a variable number of sessions (between three and eight) 2 weeks apart on the surgical site, with acceptable tolerance, sustained responses after a year and good cosmetic outcomes [19,20]. Similar results were obtained in another report combining two CO_2_ laser sessions with conventional ALA-PDT for the treatment of upper lip keratoacanthoma [21]. PDT alone has proved to be an effective approach to microinvasive SCC of the lip, also with a sustained response after two sessions [22].

Post-surgical treatment with imiquimod 5%, applied daily for 2 weeks after surgery without clear margins and once weekly thereafter, has provided a sustained response after 18 months in an elderly man [4]. This scheme had been previously proposed for the management of local recurrence of oral SCC in the setting of metastatic disease [23].

Methotrexate is a dihydrofolate reductase inhibitor with an antiproliferative effect [24]. Intralesional methotrexate (IL-MTX) has been put forward as a convenient option for the management of surgically complex lip SCC. A retrospective cohort study of the pre-surgical administration of single-dose IL-MTX in SCC pointed out its effectiveness in tumoral volume reduction (a mean reduction of 0.52 cm^2^) and found that this effect was greater on lip tumours, making it an optimal treatment in that location [24]. It suggested that more complex surgeries were avoided when lesions greater than 2 cm were treated [24]. A prospective study on ten lip SCC treated with two doses of IL-MTX found a reduction in tumoral size in every patient, with complete resolution of the smallest lesions, without AEs [25].

Intraarterial chemotherapy has been administered in some head and neck cancers as a palliative treatment. Its role in lip SCC is more limited. A study showed that cycles of infusion of methotrexate into the external temporal artery (50 mg daily for 8 days, followed by 25 mg weekly) achieved significant responses in 20 out of 21 patients with commissure SCC, complete in 62% of cases, without major AEs [26]. Neoadjuvant intraarterial cisplatin also had a satisfactory outcome in a reported case, with clinical complete regression and no recurrences 4 years after resection of the affected site [27].

## 3. Topical Immunotherapy in Cutaneous Cancer of the Eyelids

Eyelid malignant neoplasias represent up to 5–10% of all cutaneous cancers [28]. Tumours in this location can potentially involve the eye and/or nearby neural structures, causing loss of visual acuity or even lethal complications. At the same time, they constitute a therapeutic challenge, as relatively small tissular defects can readily compromise eyelid function, limiting oncologic surgery in many cases, with more frequent recurrences [29]. Radiotherapy is an efficient alternative with cosmetically acceptable results, but it can result in eye redness, dryness, glaucoma, or cataracts if excessive doses or multiple cycles are administered. These peculiarities make topical immunotherapy an especially valuable therapeutic option in this context.

### 3.1. Basal Cell Carcinoma

BCC accounts for up to 90% of eyelid tumours [29], and approximately 20% of BCCs arise on the eyelid [30]. While Hedgehog inhibitors have proven their utility in periocular BCC, being effective in more than 50% of cases, they are often poorly tolerated due to common class AEs, such as muscle spasms, ageusia, alopecia, or weight loss [29].

While not approved for its periocular use, the effectiveness and tolerability of imiquimod 5% has been frequently described in this setting [30,31,32,33,34,35]. A series of 24 cases treated with imiquimod five times a week for a variable period of two to four months showed a histologic CR rate of 89.5% after three months and 84.2% after three years. When stratifying by diameter, every lesion smaller than 1 centimetre responded completely [30].

There exist other smaller series in the literature using varying imiquimod courses presenting global CR rates of 80–100% after follow-up periods oscillating between 6.5 months and 7 years [31,32,33,34,35,36,37]. Globally, nodular BCCs show higher recurrence rates than their superficial counterpart. However, a study including 19 periocular nodular BCCs treated with varying courses of imiquimod found histological CR rates of 84.2% after 3 years. The two lesions in the series that presented a partial response (PR) were larger than 1 cm [30]. It was concluded that, in those tumours larger than 1 centimetre, treatment courses of 6 weeks convey a worse prognosis when compared to longer ones. The best results in the literature were obtained after 12 weeks of one daily application, with CR rates of 76%, while they were slightly inferior with the same regimen after 6 weeks (CR: 71%) [30]. Patients were instructed to apply imiquimod using a swab and to avoid direct conjunctival contact. Prophylactic artificial tears were prescribed. There were three patients who interrupted treatment because of AEs. In this series, 95% presented with conjunctivitis, 84% with keratitis, 57% with tearing, and 37% with ectropion. They were all self-limited [30]. These rates of ocular involvement differ from those of other reports: a review found that conjunctivitis was stated in only 9 out of 81 patients receiving periocular imiquimod for different pathologies [38]. However, redness was found in 89% of patients, without specifying whether cutaneous or ocular [38]. Rarer more serious AEs, such as infectious keratitis, corneal oedema, or ectropion, were also reported once each [38].

5-FU has also been employed in this setting. It proved to be non-inferior to imiquimod in the treatment of superficial BCC [39]. A comparative retrospective study of imiquimod 5% used twice daily on alternate days versus 5-FU 1% used twice daily on 30 eyelid BCCs, of which 53.3% were nodular, 36.7% were superficial, and 10% were basosquamous, showed a CR in 62% and 57.1% of cases, respectively. 5-FU achieved however a greater number of PRs and less non-responses (28.5% and 14%, respectively, versus 18,8% of each with imiquimod) [40]. Imiquimod produced erythema more frequently. There were five cases of keratitis punctata with imiquimod and two with 5-FU [40].

PDT is another documented therapeutic resource for periocular BCC. A review including 75 patients treated with red light PDT found an initial CR rate of 77% [28]. A sustained response after a mean period of 23 months was attained by 55% of patients. MAL elicited better results than ALA in this review (CR of 87% versus 42%) [28]. The number of performed sessions varies between publications [41,42,43,44,45]. Usually, two or three cycles are required [41,42,45]. Ocular protective shields and conjunctival anaesthesia are used [41,42,45]. Moreover, another instance describes three laser PDT sessions with ALA 10% on the surgical site of eight infiltrative BCCs after limited surgeries. After 2.8 years of follow-up, no recurrences were detected [44].

Interferons are a naturally occurring family of pleiotropic cytokines with antitumoral effects [46]. Intralesional interferon has been administered to BCCs with an effectiveness of 67–80% [46,47]. This modality was used in a series of 11 periocular BCCs, 7 of them with local aggressivity features. Interferon was injected three times per week for 2 weeks. After a year of follow-up, there were no documented recurrences, and only one patient experienced associated flu-like symptoms [47]. Ophthalmic interferon has been applied in clinical practice to treat multiple ocular surface lesions, such as warts, SCC, Kaposi sarcoma, or even melanoma [46]. The usual dosage is an eyedrop (1 million UI/mL) four times a day, over 12–16 weeks. This regimen was given in a case of eyelid margin BCC, which did not recur after 3 years of follow-up [46].

Local chemotherapy can also attain positive outcomes. A study described three periocular BCCs and a Kaposi sarcoma treated with intralesional bleomycin, regressing completely after a variable number of four to eight cycles over 6 to 12 months. Remission was maintained for up to 3 years [48]. Electroporation was applied in an instance to increase the permeability to chemotherapy (bleomycin and cisplatin), both intravenous and intralesional, of 12 eyelid BCCs, 9 of which were local recurrences [49]. Histological clearance was shown in eleven of them. This method can be useful to minimize local AEs when treating aggressive BCCs.

### 3.2. Squamous Neoplasias

SCC, along with SCC in situ (SCCis) and AK, is the second most common malignancy in this location, usually involving the lower eyelid and the margin [29]. Topical 5-FU constitutes a treatment option for eyelid AK, with only limited local AEs [50]. It is a common treatment for ocular surface squamous neoplasia [51]. Its use in 14 patients is described in this setting (13 AKs and 1 Bowen disease; 9 of them on the eyelid margin), applied in cycles of 2 weeks which were repeated in the case of recurrence. After one to three repetitions, disease control was achieved after a mean follow-up period of 3 years [51]. Only two mild ocular AEs (keratitis punctata and chemosis) occurred, independently of the location of the lesion with respect to the margin [51]. There are further isolated case reports on the effectiveness of 5-FU in eyelid SCCis [52].

Imiquimod is approved for its use in AKs, and showed a 71% success in the treatment of small invasive SCC in other locations [50]. A retrospective study obtained a 77% success in 47 periocular tumours (37 AKs, 7 SCCis, and 3 BCCs) using imiquimod thrice weekly for 4–6 weeks. However, 32% of patients developed conjunctivitis, and 20% required discontinuation [53]. Also, the resolution of a case of eyelid microinvasive SCC was achieved after two standard cycles of imiquimod 3.75% [54]

Promising results were also reported with PDT [28]. Four case reports [28,55,56,57,58] (one AK [55], two SCCis [57,58], and an SCC [56]) showed complete tumoral regression after a minimal follow-up of 6 months. Except for the SCC, which was clinically followed-up after a single cycle, every lesion received two sessions, and the response was histologically proven. In cases of more invasive tumours, PDT with intravenous photosensitizers has been successful. This procedure has been applied to conjunctival SCC in three instances, using verteporfin. An elderly patient with a temple SCC near her eyelids was also reported to be treated with PDT after the administration of intravenous hiporfin, with subsequent biopsied tumoral resolution after 6 weeks [20].

Other topical therapies such as diclofenac were used in eyelid AKs, with a partial effect. In a series on four patients, lesions regressed after 4 months of treatment, but they recurred in two cases after 4 and 7 months, respectively [59]. In spite of this, it caused mild irritation in only one patient and had high acceptability, making it a good candidate for maintenance treatments [59].

Reports on intralesional treatment of periocular SCC are anecdotical. A tumoral volume reduction of 69% was attained after 13 injections of IL-MTX in a giant temple SCC adjacent to the eyelid [60]. A case of CR of a clinically diagnosed lower eyelid SCC after a single administration of intralesional and perilesional cidofovir was described [61]. Intralesional 5-FU was ineffective in a documented case of eyelid keratoacanthoma [62].

### 3.3. Lentigo Maligna

Lentigo maligna (LM) makes up to 10–26% of all head and neck melanomas [63]. Due to its usual wide local extension and invasive potential, its management poses a challenge. Up to 3.3% of LM diagnosed at 45 years of age progresses to invasive melanoma, a higher percentage compared to 1.2% of those diagnosed at 65 years of age [38]. Mohs micrographic surgery (MMS) remains the gold-standard treatment, with recurrence rates of 2% after 10 years. The majority of MMSs performed on LM result in surgical margins of 5 mm; however, up to 31% of cases need greater margins for complete excision [63]. Of these more complex cases, 19% involve the eyelid [38]. Thus, alternate strategies are particularly useful in this setting to avoid aggressive surgical procedures.

Experience with imiquimod in the treatment of LM has been documented, with clinical CR rates ranging from 77% to 93% at different times of evaluation [38,63,64]. The histological responses are about 75% [38,63]. Different regimens have been used, most frequently five to seven applications a week for 12 weeks, over the lesion and 1–2 cm beyond its visible margins [65]. A study suggested more favourable outcomes when a cumulative number of 60 applications is reached, independently of timing [65]. A literature review on imiquimod for the treatment of eyelid LM found a clinical effectiveness of 86%, while histopathologic confirmation was obtained in only 56% of cases. These findings are equivalent to the previously described 77% CR rate according to the authors [38]. Treatment cessation due to local EAs was needed in 9.9% of cases [38]. A small series of five eyelid LM treated with imiquimod on varying timings found two CRs, which both followed an intense inflammatory reaction, and three PRs, out of which two had mild adverse effects [38]. These observations match those of former research on LM, which found that 97% of responders, and 50% of non-responders, suffered strong inflammatory reactions [63].

Intralesional interferon was also described in LM. A series of 11 cases, including a periocular and a perioral tumour, showed complete clinical clearance after a mean number of 19 doses (range: 12 to 29) in 2 to 5 months in 9 cases [66]. A histological CR was proven in four of them (including those apparently non-responsive, showing numerous melanophages) [66]. The total number of doses was dependent on the lesional size [66]. The toxic effects (mainly flu-like symptoms) were minor and transitory [66]. Another case report demonstrated the utility of this modality in complex periocular tumours, achieving a complete histological clearance in a LM involving both eyelids and conjunctiva after injecting 39 million UI into one site on the upper eyelid and three on the lower one [67].

## 4. Conclusions and Future Directions

Local therapy is an efficient, convenient, and safe option for the management of the most common types of cutaneous cancer in both perioral and periocular areas, proven to be particularly useful when conventional approaches, such as surgery or radiotherapy, are not suitable options due to their functional and aesthetic impact on these locations. Multiple treatment lines have been described in these settings (Tables S1 and S2). The implementation of their use will hopefully lead to increasing knowledge and experience about the potential and particularities of each treatment, enabling the therapeutical optimization of particular cases. Moreover, agents such as checkpoint inhibitors, which have recently marked a shift in the paradigm of the management of both melanocytic and non-melanocytic advanced cutaneous cancers, may be locally used in the future. While still widely used as systemic treatments, there are currently ongoing trials around their intralesional administration in different types of cutaneous cancers [68].

This review provides a comprehensive scope of the available therapeutic approaches in diverse types of cutaneous neoplasias in the aforementioned locations. However, the referenced evidence is heterogeneous, and large randomized controlled trials are lacking, mainly due to the relative lesser frequency of these tumours and surgery being the gold-standard treatment. In addition, a direct comparison of results was not performed, hindering their clinical applicability. In spite of this, these modalities suggest a promising horizon in the treatment of skin cancer on complex anatomical sites.

## Supplementary Materials

The following supporting information can be downloaded at: https://www.mdpi.com/article/10.3390/cancers15205018/s1, Table S1: Studies using topical and intralesional therapy for perioral cutaneous cancer (including actinic cheilitis, lip squamous cell carcinoma and perioral lentigo maligna); Table S2: Studies using topical and intralesional therapy for periocular cutaneous cancer (including basal cell carcinoma, actinic keratosis squamous cell carcinoma and lentigo maligna).
