# Supplementary material for: Topical and Intralesional Immunotherapy for the Management of Skin Cancer in Special Locations: Lips and Eyelids
Source: Cancers (Basel). 2023 Oct 17;15(20):5018. doi: 10.3390/cancers15205018 (PMC10604909; doi:10.3390/cancers15205018)
Supplement: Supplementary file 1 [file cancers-15-05018-s001.zip › cancers-2614739-supplementary.pdf]

Supplementary materials:

Table S1: Studies using topical and intralesional therapy for perioral cutaneous cancer (including actinic cheilitis, lip squamous cell carcinoma and perioral lentigo maligna).

Topical therapy in perioral cutaneous cancer									
**Topical therapy for actinic cheilitis**									
**Therapeutic modality**	**References**	**Type of study**	**Nº of patients**	**Initial regimen**	**Clinical clearance**	**Histological clearance**	**Mean follow-up (months)**	**Recurrence**	**Comments**
**IMI**	Husein-El Ahmed et al, 2019[1,5,9,10]	Clinical trial	10	IMI 5% thrice a week for 4 weeks	CR: 50%<br>PR: 40%<br>NR: 10%	N/A	8.7	0	Patient satisfaction index: 5.4/10.
	Smith et al, 2002[5,6,9]	Retrospective study	15	IMI 5% thrice a week for up to 6 weeks	CR: 100% (at 4 weeks after end)	N/A	7.3	0	Additional treatment duration may not be more effective
	McDonald et al, 2010[6,10]	Retrospective study	5	IMI 5% 5 times a week for up to 6 weeks	CR: 100%	CR: 100%	1	-	-
	Spyridonos et al, 2014[6]	Prospective study	8	IMI 5% once daily for 5 weeks	CR: 62,5%<br>PR: 37,5%	N/A	6	-	-
**5-FU**	Epstein, 1977[5,9]	Prospective study	12	5-FU 5% 3-4 times a day	CR: 100%	N/A	22	16.7%	-

				for a mean of 12 days					
	Robinson et al, 1989[5,9]	Prospective study	10	5-FU 5% thrice a day for 4 weeks	N/A	N/A	50	50%	Recurrences proven by biopsy
	Warnock et al, 1981[9]	Prospective study	6	5-FU 1% Twice a day for 2-3 weeks	CR: 100%	PR: 83% NR: 16%	1-2	-	Biopsy taken after post-treatment inflammation subsided
**PDT**	Bakirtzi et al, 2021[9]	Systematic review (13 studies)	241	Daylight, ALA or MAL-PDT over 1-6 sessions	CR: 66.7%	CR: 49.5%	14.8	14.1%	Discontinuation rate: 5.9%
	Lai et al, 2020[10]	Systematic review (18 studies)	257	Daylight, ALA or MAL-PDT over 1-6 sessions	CR: 68.9% PR: 27.6& NR: 8.3%	CR: 42.5%	11.1	12.6%	Discontinuation rate: 6.2%
**Daylight PDT**	Fai et al, 2015[9]	Prospective study	10	2 sessions of daylight MAL-PDT 1-2 weeks apart	CR: 70%	N/A	7	28.6%	-
	Lai et al, 2020[10]	Systematic review (3 studies)	23	Daylight MAL-PDT over a mean	CR: 82.6% PR: 9.1%	CR: 100%	20.5	13%	-

				of 2.5 sessions					
	Andreadis et al, 2020[13]	Prospective study	22	2 sessions of daylight MAL-PDT a week apart	CR: 81.8% PR: 9.1%	N/A	12	11.1%	Painless in 80% of cases. 2 patients lost to follow-up
	Levi et al, 2019 [9,14]	Prospective study	11	Daylight MAL-PDT over a mean of 2 sessions	CR: 91%	CR: 100% (45% biopsied)	30	10%	-
**Laser-mediated PDT**	Bakirtzi et al, 2021[9]; Lai et al, 2020[10]	Systematic reviews (2 studies)	33	ALA-dye 595 and MAL-Er:YAG up to 3 sessions	CR: 75.8% PR: 10.5%	N/A	7.5	6.1%	-
**PDT + imiquimod 5%**	Sotiriou et al, 2011 [10,16]	Prospective study	34	2 MAL-PDT 2 weeks apart followed by IMI 5% thrice a week for 4 weeks	CR: 90% PR: 10%	CR: 73% (after 12 months)	12	11.1%	4 patients lost to follow-up
**IM**	Husein-El Ahmed et al, 2019 [1,5,9]	Clinical trial	10	IM 0.015% on 3 consecutive days	CR: 40% PR: 30% NR: 30%	N/A	6	0	Non-inferior to IMI 5% and shorter posology
	Flórez et al, 2017 [5,9,17]	Case series	7	IM 0.015% on 3 consecutive days	CR: 42.9% PR: 57.1%	N/A	2	-	Partial responses represented an improvement

									over 75% from basal.
	Rossini et al, 2021 [5,18]	Prospective study	14	IM 0.015% on 3 consecutive days	CR: 0% PR: 100%	CR: 0%	2	-	While objective clinical or histological complete response was not achieved, subjective improvement was noted in all patients.
	Lai et al, 2020[10]	Systematic review (5 studies)	25	IM 0.015% on 3 consecutive days	CR: 52% PR: 38.1% NR: 30%	N/A	5.2	25%	-
**Diclofenac**	Husein-El Ahmed et al, 2019 [1,5,9]	Clinical trial	10	Diclofenac 3% twice daily for 6 weeks	CR: 20% PR: 40% NR: 40%	N/A	6	0	-
	Lima et al, 2010[5,9]	Prospective study	27	Diclofenac 3% twice daily for 1 to 6 months	CR: 44% PR: 56%	CR: 0% (15 patients)	4.7	-	Those who were biopsied were those with clinical partial response
	Gonzaga et al, 2018[5,9]	Prospective study	31	Diclofenac 3% thrice	CR: 66.7% PR: 20%	N/A	6	0	12 lost to follow up.

				daily for 3 months					5 interrupted treatment due to adverse effects. 2 prolonged 30 additional days
	Bakirtzi et al, 2021[9]	Systematic review (4 studies)	62	Diclofenac 3% twice daily for 1 to 6 months	CR: 45.2%	N/A	4.4	6.5%	-
**Topical and intralesional therapy in invasive squamous cell carcinoma of the lip**									
**Therapeutic modality**	**References**	**Type of study**	**Nº of patients**	**Initial regimen**	**Clinical clearance**	**Histological clearance**	**Mean follow-up**	**Recurrence**	**Comments**
**Laser-PDT**	Yan et al, 2020[19]	Case report	2	Superficial curettage followed by 3 and 8 sessions of ALA-PDT every 2 weeks with a 635 nm laser	CR: 100%	N/A	12 and 24 months	No	-
	Wang et al, 2022[20]	Case report	1	Single-dose Intravenous Hiporfin at doses of 5	Yes	Yes	53 days	No	600 ul of 2 mg/ml hipofin solution injected intralesionally 3

				mg/kg followed by two irradiations (at 48 and 72 h) with 630 nm laser.					hours before each irradiation
	Liang et al, 2020[21]	Case report	1	4 sessions of CO^2 laser + ALA-PDT every week	Yes	N/A	3 years	No	-
**PDT**	Fargnoli et al, 2015[22]	Case report	1	2 sessions of MAL-PDT	Yes	Yes	2 years	No	-
**IMI**	Pentangelo et al, 2021[4]	Case report	1	IMI 5% daily for 2 weeks and then once a week.	Yes	N/A	2 years	No	-
**IL-MTX**	Salido-Vallejo et al, 2016[24]	Retrospective cohort	43 (5 in lower lip)	A single dose of intralesional MTX (syringes of 25 mg/ml) previous to surgery	No	N/A	N/A	N/A	Mean volume reduction of 0.52 cm^2 before surgery. IL-MTX shown to avoid complex surgeries in lip SCC.

	Bergón-Sendín et al, 2019[25]	Prospective study	10	2 doses of 20 mg of IL-MTX	CR: 30%<br>PR: 70%	N/A	N/A	N/A	Mean reduction of 57.3% of major diameter and 68.2% of minor diameter
**Intraarterial chemotherapy**	Wu et al, 2014[26]	Retrospective study	21	Initial intraarterial daily 50 mg of MTX (mean 8 days) followed by weekly 25 mg of MTX (mean 10 weeks)	CR: 62%<br>PR: 33%<br>NR: 5%	57.1%	69 months	38.4%	12 of 13 patients with clinical CR showed no histological evidence of tumour. Minimal systemic effects
	Yokota et al, 2017[27]	Case report	1	Intraarterial weekly 100 mg/m2 of cisplatin for 5 weeks	Yes	Yes	5 years	No	Minimal systemic effects
**Topical and intralesional therapy in perioral lentigo maligna**									
**Therapeutic modality**	**References**	**Type of study**	**Nº of patients**	**Initial regimen**	**Clinical clearance**	**Histological clearance**	**Mean follow-up**	**Recurrence**	**Comments**
**Intralesional interferon**	Cornejo et al, 2000[66]	Case series	1 (perioral location)	3 million UI of	Yes	N/A	25 months	No	-

				intralesional interferon-alpha thrice weekly up to 29 doses					

ALA: aminolevulinic acid. **CR**: complete response. **IL-MTX**: intralesional methotrexate. **IM**: ingenol mebutate. **IMI**: imiquimod. **MAL**: methyl-aminolevulinic acid. **NR**: non-response. **PDT**: photodynamic therapy. **PR**: partial response. **5-FU**: 5-fluorouracil.

Table S2: Studies using topical and intralesional therapy for periocular cutaneous cancer (including basal cell carcinoma, actinic keratosis squamous cell carcinoma and lentigo maligna).

Topical and intralesional therapy in periocular cutaneous cancer									
**Topical and intralesional therapy for periocular basal cell carcinoma**									
**Therapeutic modaity**	**References**	**Type of study**	**Nº of patients**	**Initial regimen**	**Clinical clearance**	**Histological clearance**	**Mean follow-up**	**Recurrence**	**Comments**
**IMI**	De Macedo et al, 2015[30]	Prospective study	24	IMI 5% 5 times a week for 8-16 weeks.	CR: 89.5%	CR: 89.5%	39.5 months	5.9%	Nodular subtype. 5 patients interrupted treatment. Histological response of 100% after 3 years for lesions smaller than 1 cm.
	Prokosch et al, 2011[31]	Prospective study	5	IMI 5% 5 times a week for 6 weeks	CR: 100%	N/A	7 years	0%	Nodular subtype. 1 patient interrupted treatment.

	García-Martín et al, 2011[32]	Clinical study	15	IMI 5% 5 times a week for 6 weeks	CR: 100%	CR: 100%	24 months	0%	Nodular subtype. Worse tolerated than radiotherapy but better functional results.
	Carneiro et al, 2010[33]	Case series	8 (10 lesions)	IMI 5% 5 times a week for 8-16 weeks	CR: 80%	CR: 80%	11.8 months	0%	Nodular subtype
	Leppäla et al, 2007[34]	Case series	4	IMI 5% 5 times a week for 6 weeks	CR: 100%	CR: 100%	26 weeks	0%	Nodular subtype
	Blasi et al, 2005[35]	Case series	2	IMI 5% 3 times a week for 12 and 8 weeks, respectively	CR: 100%	N/A	1 year	No	Nodular subtype
	Choontanom et al, 2006[36]	Case series	5	IMI 5% 5 times a week for 6 weeks	CR: 80%	N/A	3 years	0%	Nodular subtype. Mean diameter: 10 mm
	Ross et al, 2010[37]	Case series	2	IMI 5% 5 times a week for 6 weeks	CR: 100%	N/A	12 and 9 months	No	-
**5-FU**	Singh et al, 2021[40]	Retrospective study	14	5-FU 1% Twice daily for up to 6	CR: 57.1% PR: 28.5% NR: 14%	N/A	12 months	N/A	53.3% Nodular, 36.7%

				months until response	(after 12 months)				superficial, 10% basosquamous.
**PDT**	Cerman et al, 2015[28]	Systematic review	75	Variable number of sessions (typically 2-3; range 1-12)	CR: 77%	N/A	23 months	29.3%	MAL had better CR rates than ALA (87% vs 42%)
**Laser-PDT**	Li et al, 2019[44]	Case series	8	PDT using 635 nm laser and 5-ALA 10% as a photosensitizer	CR: 100%	CR: 100%	2.8 years	0%	At least an infiltrative BCC is mentioned
**Local interferon**	Leis-Dosil et al, 2014[46]	Case report	1	Interferon alpha ophthalmic 4 droplets a day (1 million UI per droplet) for 4 months	Yes	N/A	3 years	No	-
	Fenton et al, 2002[47]	Retrospective study	11	Intralesional interferon alpha, 3 million units thrice a week for 2 weeks (total: 18 million)	CR: 100%	CR: 100% (8 patients)	12 months	No	Out of 11 tumours, 10 had been previously treated with surgery with affected margins, and 7 belonged to high-risk histological subtypes.

**Local chemotherapy**	Meyer et al, 2015[48]	Retrospective study	3	Intralesional bleomycin 1 UI/ml together with lignocaine 2% in a ratio of 4:1, 4 to 8 cycles in 6 to 12 months	CR: 100%	N/A	4 years	No	-
	Kis et al, 2019[49]	Case series	12	1 to 5 electroporation sessions using 250-1000 UI/cm² of intralesional bleomycin (3 patients) or 15000 UI/m² (9 patients)	CR: 100%	CR: 100% (11 patients)	25.3 months	0%	3 cases of ectropion due to post-procedure scar needed surgical correction.
**Topical and intralesional therapy for periocular squamous cell neoplasias**									
**Therapeutic modality**	**References**	**Type of study**	**Nº of patients**	**Initial regimen**	**Clinical clearance**	**Histological clearance**	**Mean follow-up**	**Recurrence**	**Comments**
**5-FU**	Couch et al, 2012[51]	Retrospective study	14 patients (13 AKs and 1 Bowen)	5-FU 5% Twice daily for 2 weeks, one initial cycle and repetition if partial response, recurrence	CR: 71,4%%<br>PR: 28,5%	N/A	36.6 months	30%	-

	Sharkawi et al, 2011[52]	Case report	1 (Bowen)	5-FU 5% twice daily for 6 weeks	Yes	N/A	3 years	No	-
**IMI**	Cannon et al, 2011[53]	Retrospective study	47 patients (37 AK, 7 Bowen, 3 BCC)	IMI 5% thrice a week for 4-6 weeks	CR: 72.3%	N/A	16 weeks	0%	9 patients needed interruption of treatment, starting again in 6. One patient lost to follow-up. Complete resolution of adverse effects
	Toso et al, 2022[54]	Case report	1	IMI 3.75% daily over two cycles of 2 course of 2 weeks each (total: 8 weeks)	Yes (complete)	N/A	6 months	No	-
**PDT**	Rossi et al, 2004[56]	Case report	1 SCC	A single session of 20% ALA-PDT irradiated with an incoherent light source	Yes (complete)	N/A	6 months	No	-
	Calista et al, 2008[58]	Case report	1 in situ SCC (Bowen)	Two sessions of 16% ALA-PDT after curettage, irradiated with	Yes (complete)	Yes (complete)	30 months	No	-

				diode lamp, one week apart					
**Laser-PDT**	Sunohara et al, 2012[57]	Case report	1 Bowen	Two sessions of ALA-PDT irradiated with pulsed dye laser	Yes (complete)	Yes (complete)	1 month	No	-
**PDT + imiquimod 5%**	Toledo-Arberola et al, 2012[55]	Case report	1 AK	2 red-light PDT sessions 2 weeks apart with 16% ALA followed by IMI 5% thrice a week for 4 weeks	Yes (complete)	Yes (complete)	N/A	N/A	Immediate response is described after finishing treatment
**Diclofenac**	Batra et al, 2012[59]	Case series	4 AK	Diclofenac 3% gel twice daily for 1 to 4 months	CR: 100%	N/A	8.3 months	50%	-
**Other intralesional treatments**	Carriere et al, 2020[60]	Case report	1 SCC	13 cycles of IL-MTX (first 3 cycles of 25 mg, followed by 10 cycles of 50 mg), administered every 2 weeks	Yes (partial)	N/A	6 months	N/A	Sustained tumoral volume reduction of 69%
	Calista et al, 2002[61]	Case report	1 SCC	A single dose of 7.5 mg of intralesional cidofovir	Yes (complete)	Yes (complete at 12 months)	24 months	No	-

	Requena et al, 1990[62]	Case report	1 SCC	4 weekly cycles of 50 mg of intralesional 5-FU, in addition to topical 5-FU 5% once or twice daily and radiotherapy	No	No	N/A	N/A	After therapeutic failure, the patient required extensive surgery, with exenteration and no recurrence after 24 months
**Topical and intralesional therapy for periocular lentigo maligna**									
**Therapeutic modality**	**References**	**Type of study**	**Nº of patients**	**Initial regimen**	**Clinical clearance**	**Histological clearance**	**Mean follow-up**	**Recurrence**	**Comments**
**IMI**	Neumann et al, 2023[38]	Systematic review	21	Variable regimens of IMI 5% ranging from thrice weekly to once daily during 1 to 24 months; alone (13) or combined with other modalities (6 surgery and/or cryotherapy, 2 topical	CR: 85.7% PR: 14.2%	CR: 92% (12 patients)	21.9 months	No	-

				chemotherapy for conjunctival involvement)					
	Misiak-Galazka et al, 2022[65]	Case report	1	2 cycles of IMI 5% 5 times a week during 5 weeks, 2 months apart	Yes (complete)	N/A	2 years	No	First cycle interrupted due to intense inflammation, the same duration was implemented in the second cycle
**Local interferon**	Cornejo et al, 2000[66]	Case series	1 (periocular location)	6 million UI of interferon-alpha intralesionally thrice weekly for 5 weeks (15 doses)	Yes (complete)	N/A	19 months	No	-
	Carucci et al, 2000[67]	Case report	1 (involving upper and lower eyelid)	3 million UI of interferon-alpha intralesionally in each eyelid, thrice weekly, for a total of 39 million UI in each site.	Yes (complete)	Yes (complete)	N/A	No	Previously treated with excisional surgery. Follow-up time not specified.

AK: actinic keratosis. **ALA**: aminolevulinic acid. **BCC**: basal cell carcinoma. **CR**: complete response. **IL-MTX**: intralesional methotrexate. **IMI**: imiquimod. **MAL**: methyl-aminolevulinic acid. **NR**: non-response. **PDT**: photodynamic therapy. **PR**: partial response. **SCC**: squamous cell carcinoma. **5-FU**: 5-fluorouracil.
